# Near-term climate risks and sunlight reflection modification: a roadmap approach for physical sciences research

**DOI:** 10.1007/s10584-022-03446-4

**Published:** 2022-10-17

**Authors:** Kelly Wanser, Sarah J. Doherty, James W. Hurrell, Alex Wong

**Affiliations:** 1 SilverLining, Washington, DC USA; 2 Department of Atmospheric Sciences, Cooperative Institute for Climate, Ocean and Ecosystem Studies, University of Washington, WA Seattle, USA; 3 Department of Atmospheric Sciences, Colorado State University, Fort Collins, CO USA; 4 Pardee RAND Graduate School, Santa Monica, CA USA

**Keywords:** Climate risk, Climate intervention, SRM, Solar radiation modification, Marine cloud brightening, Stratospheric aerosol injection, Geoengineering

## Abstract

Current impacts and escalating risks of climate change require strong and decisive action to reduce greenhouse gas (GHG) emissions. They also highlight the urgency of research to enhance safety for human and natural systems, especially for those most vulnerable. This is reflected in two recent US National Academies of Science, Engineering, and Medicine studies that recommended a national focus on advancing our understanding of how to manage urgent current and future climate risks, and the study of approaches for increasing the reflection of sunlight from the atmosphere to reduce global warming, a process referred to as sunlight reflection modification (SRM). Here, we build on these recommendations by proposing a roadmap approach for the planning, coordination, and delivery of research to support a robust scientific assessment of SRM to reduce near-term climate risks in a defined timeframe. This approach is designed to support the evaluation of SRM as a possible rapid, temporary, additive measure to reduce catastrophic impacts from anthropogenic climate change, not as a substitute for aggressive GHG mitigation. Assessing SRM is proposed to be undertaken in the context of climate hazard risks through 2050, weighing the impacts associated with likely climate change trajectories against scenarios of possible SRM implementations. Provided that research is undertaken openly and that scientific resources are made widely available, the transparency of the process and the evidence generated would contribute to the democratization of information, participation by diverse stakeholders, more informed decision-making, and better opportunities for all people to weigh SRM options against climate change risks.

## Introduction

Current impacts and escalating risks of climate change require strong and decisive action to reduce global emissions of greenhouse gases (GHGs) (Intergovernmental Panel on Climate Change (IPCC 2022a; IPCC 2021a; IPCC 2021b; Blunden and Boyer 2020). They also highlight an urgent need for research to enhance the safety of human and natural systems, especially for those most vulnerable (IPCC 2022b; National Academies of Sciences, Engineering, and Medicine (NASEM) 2021c; Environmental Protection Agency 2021).

In particular, better information on climate risks is needed because the Earth’s climate will warm substantially by 2050 under all emission scenarios considered by IPCC (IPCC 2021b; Mauritsen and Pincus 2017; Samset et al. 2020; Lenton et al. 2019). Such warming increases the near-term (i.e., 10–40 year) risk of climate impacts (Arnell et al. 2019) and enhances risk of major changes in natural systems that substantially increase warming (i.e., feedbacks) and/or impacts (i.e., “tipping events”). Recent observations of temperature extremes in polar regions and instabilities in permafrost, ice sheets, terrestrial forests, and circulation systems indicate these risks may be significant (Petit et al 2021, Fewster et al. 2022, Boulton et al. 2022, Boers 2021).

In this context, in March 2021 the US National Academies of Science, Engineering, and Medicine (NASEM) published a pair of studies making recommendations for the direction of US climate research. One urged the USA to focus on providing insights that help prepare for and avoid the worst potential consequences of climate change (NASEM 2021b). Another recommended the USA evaluate approaches for increasing the reflection of sunlight from particles and clouds in the atmosphere to rapidly reduce global warming, otherwise known as sunlight reflection modification (SRM^1^) (NASEM 2021c).

SRM is a class of proposed approaches identified by the scientific community as the most promising active intervention for rapidly reducing global warming (Shepherd et al. 2009; NASEM 2015; NASEM 2021c). SRM can be accomplished either by increasing the amount of sunlight reflected by atmospheric aerosols and clouds or by increasing the amount of outgoing long-wave radiation from Earth by changing cloud properties. The NASEM reports expansively covered the arguments for and against SRM research, which remains controversial among some scientists and stakeholders. Nonetheless, SRM might be considered as part of the portfolio of societal responses to the present and growing risks of climate change. As such, robust information is needed on the efficacy and risks of SRM approaches and on how they would alter climate impacts in different regions under various scenarios (Jabbour and Flachsland 2017; NASEM 2021c) and projections for warming against projected near-term climate impacts and tipping event risks.

While assessing available science is critical to equip policymakers and the public with information to inform decision-making (Watson 2012; Biniaz and Bodansky 2020), it is only possible if there is a robust body of science. This poses challenges for emerging areas of research, such as SRM, where scientific evidence is scant. The central scientific problem of SRM is also central to climate research: understanding the influence of aerosols and clouds, and their interactions, on the atmosphere and climate (IPCC 2021a). Generating sufficient new knowledge and advancing required existing research areas and capabilities, particularly within a defined timeframe, requires a “roadmap” approach that integrates and coordinates diverse research activities toward achieving a shared set of objectives.

The following sections describe a method for building a roadmap and iterating it to assess near-term climate risks and SRM.

## Research scope

Research required to assess the physical aspects of near-term climate risks and SRM approaches include modeling, analytics, and observation of relevant natural systems to compare the likely effects of interventions versus projected warming as well as the identification of thresholds that might be relevant points of intervention for safety (Finkel 2011; Fast Track Action Committee on Earth System Predictability Research and Development 2020). Generating this information requires focused research as well as substantial investments in scientific capabilities for climate observation and prediction.

This required body of research is highly interdisciplinary (Pörtner et al. 2021) and centered in atmosphere and Earth system sciences. It also requires research in related natural sciences and human systems sciences for studying impacts and other aspects of safety and sustainability (NASEM 2021c). Recent studies in Earth system and atmospheric sciences have demonstrated the benefits of leveraging analytical tools from other disciplines, including applied mathematics and statistics (Smid and Costa 2018; Majumdar et al. 2021), complex systems, and uncertainty research. Engineering research is critical for understanding materials, platforms, and implementation regimes for SRM approaches.

### Anthropogenic analogs

The mechanisms by which SRM could be used to cool climate have been observed through the effects of both natural and anthropogenic emissions. Particles (namely, aerosols) from anthropogenic sources, such as factories and power plants, increase the reflection of sunlight from the atmosphere both directly (i.e., sunlight scattering off of particles) and indirectly (i.e., where they enhance the reflectivity of clouds). The collective effect of aerosol pollution was recently assessed as virtually certain to be negative (a cooling effect), exerting a climate forcing of − 1.1 Wm^2^ (− 1.7 to − 0.4 Wm^2^), counteracting about one-third of the forcing by GHGs. Notably, this is the most uncertain of the anthropogenic climate forcing influences (IPCC 2021a).

Because SRM research centers on understanding influences on atmospheric processes that drive climate and analyzing climate impacts and uncertainties, much of the needed research is dual purpose, with the potential to both accelerate a broad-based understanding of climate while also improving the management of climate risks (NASEM 2015; Wood et al. 2017; Kremser et al. 2016; Portier 2010).

### SRM approaches

The most promising SRM approaches identified by NASEM (2021a) and others for rapidly reducing warming involve dispersing aerosols in the stratosphere via stratospheric aerosol injection (SAI), in the lower tropospheric marine boundary layer via marine cloud brightening (MCB), or into cirrus clouds in the upper troposphere via cirrus cloud thinning (CCT). The intent of these approaches is to increase the reflection of sunlight from the atmosphere (SAI and MCB) or increase Earth’s outgoing longwave radiation (CCT) through direct scattering (SAI) or by changing cloud properties (MCB and CCT). The direct and indirect effects of these approaches are determined by key atmospheric and physical processes and feedbacks, including cloud–aerosol interactions, radiative transfer, heterogeneous chemistry, and atmospheric transport. As such, the lines of research, activities, and goals are similar across SRM approaches.

## Roadmap development

Roadmaps are common elements of an integrated, interdisciplinary research approach designed to support the coordinated delivery of outputs in a defined timeframe. While they are less commonly used in climate research, where much of the work is conceptualized, resourced, and undertaken as basic science, they are often used for major climate model development or observing programs (U.S. Department of Energy 2022; Universities Space Research Association n.d.; Aschwanden et al. 2021). There is growing recognition that such a coordinated and mission-driven research approach would be beneficial to improve Earth system science and predictions in general (NASEM 2020; Waliser 2020) as well as to evaluate SRM in particular (Long 2017; Wanser 2017). The following subsections describe the major characteristics of a proposed roadmap approach to define and coordinate SRM-related research.

### Roadmap timeframe

Assessing near-term climate risks and possible interventions requires reconciling the feasibility of generating and analyzing information with the urgency of policy responses. With respect to designating a timeframe for assessment of SRM, we reference escalating climate-linked extremes (AghaKouchak et al. 2020, Fischer et al. 2021, Seneviratne et al. 2012) alongside the estimated time for reaching a global temperature increase of 1.5 ºC, which is a critical threshold for natural system hazards (i.e., within 10–15 years) (IPCC 2021b). Given this, we propose a roadmap designed to significantly reduce key uncertainties in SRM approaches in a 5-year timeframe.

### Goal definition against the roadmap timeframe

One of the most important steps in planning research designed to support a scientific assessment in a specific timeframe is to define specific goals, as these determine the required deliverables and/or states of progress to be reached during a fixed timeframe.

Assessing the feasibility of SRM approaches and their potential role in reducing near-term climate risks requires research that falls within two high-level objectives:

- **Objective 1**: Characterize the key processes through which SRM approaches operate and determine how to accurately represent these processes in models used to project global climate impacts.
- **Objective 2**: Evaluate the projected near-term impacts and risks of a range of climate change scenarios without and with different SRM scenarios and strategies.

From these objectives, key questions for research can be defined, lines of research can be identified, and goals can be established. These questions and goals should be developed in the context of scientific and technical importance and feasibility prior to considering funding constraints. We will start by looking at approaches to working toward these two objectives in more detail.

### Building a roadmap to meet objectives

#### Objective 1: Characterize key processes of SRM approaches

Research for Objective 1 requires addressing the following set of questions that are applicable to all three of the leading SRM approaches (SAI, MCB, and CCT):

- **Question (Q)1**: Can aerosols of the needed size and concentration be generated and delivered into the atmosphere with the required volume and spatial distribution?
- **Q2**: How do aerosols evolve, disperse, and influence the local atmosphere under different conditions?
- **Q3**: How can the influences of SRM aerosols versus background aerosols be distinguished under different conditions?
- **Q4**: How much global cooling can be achieved through different implementations of SRM and in different future climate scenarios?
- **Q5**: What is required to incorporate SRM processes into global and regional projections of climate, under different scenarios for future climate and ranges of natural and anthropogenic emissions?

In many cases, answers to these questions are interdependent. To address these questions and define research requirements, Table 1 provides a proposed initial roadmap that delineates goals in relevant areas of research. It is organized conceptually from the lowest-level processes through scaled analysis, to provide the information needed to accurately model global effects and impacts.

**Table 1:** Proposed roadmap for Objective 1—characterize key processes of SRM approaches

R&D category	Research activities	5-year goals
Aerosol generation and dispersal studies	**• **Technology development • Near-field aerosol dispersal observations (laboratory) and modeling	• Research-grade aerosol production systems that can deliver required size and distribution of optimum materials
Localized processes and outdoor plume studies	• Local atmosphere/cloud response to aerosols via field observations, experiments, and modeling • Controlled perturbation experiments (e.g., single-plume aerosol interaction studies) as needed to test and improve models	• Characterization of conditions and processes that drive the evolution and radiative forcing efficacy of aerosols introduced into the atmosphere • Quantification of uncertainties in responses (Q1–Q3)
Limited-area processes and environmental studies	• Modeling and observational studies of local aerosol evolution and cloud responses to the addition of aerosols • Controlled perturbation experiments (multiple plumes), as needed, to test and improve models and where possible without significant environmental impact	• Understanding of chemical and dynamical processes and (except for SAI) radiative forcing at minimum scale and duration for detecting effects (Q1–Q4) • Development of sub-grid-scale parameterizations of key processes for modeling impacts • Identification of non-radiative effects (e.g., SAI impacts on ozone and MCB impacts on rainfall) (Q2) • Identification and quantification of related uncertainties in responses (Q1–Q4)
Analog and background observational studies	• Instrument development and observation of platform integration • Baselining of atmospheric conditions • Studies of natural and anthropogenic perturbation analogs	• Atmospheric baselines established for four seasons across two hemispheres (Q3) • Adequate observations established for major analogs (e.g., ship tracks and volcanic eruptions) (Q2–Q5)
Regional and global model inputs	•Inputs to regional and global climate model representations of processes underlying SRM •Development of tools from simulating SRM in regional and global models	• Mechanisms for controlled perturbation in regional and global models • Requirements/priorities for improvement in the representation of key processes associated with SRM • Identification of observational data gaps in model instantiation and/or validation of key processes associated with SRM (e.g., chemistry baseline of the stratosphere for SAI) • Proof-of-concept simulations to test and improve the above in multiple major regional and Earth system models
Implementation analysis and operational design tools and methods	• Comparative assessment of implementation strategies • Systems design (detectability, feasibility, and outcomes) • Delivery platforms (existing and purpose-designed)	• Development of tools and proof-of-concept simulations for implementation strategies to identify performance boundaries, optimize strategies, quantify model uncertainty, and analyze detection and attribution • Preliminary operational system requirements and designs to inform feasibility studies (Q2–Q5) • Studies of information, compliance, security, and continuity requirements

Note: The correspondence of these proposed 5-year goals to the key questions (see main text) are noted in parentheses

Within each line of research, a research plan can be developed based on the following:

- Which activities are considered “critical path?”
- What areas of work could be accelerated?
- Which key questions and uncertainties can be addressed through modeling and passive observational studies (e.g., of proxies) and which require small-scale controlled release experiments?

Importantly, within specific research lines and activities, research approaches can be defined to identify thresholds in key processes or first-order effects that may rule out SRM approaches, creating early off-ramps (Diamond et al. 2021) or inflection points for changes in research focus consistent with the recommendations of NASEM (2021c). As with any research area, specific goals and associated research activities would need to be revisited with new learning, thus requiring revisions to the roadmap accordingly.

#### Objective 2: Evaluate near-term impacts and risks with and without SRM

While Objective 1 is intended to characterize key processes associated with different SRM approaches and represent SRM accurately in models and other analysis tools, a robust assessment of future climate impacts and risks, both with and without SRM, requires projecting, predicting, and analyzing future trajectories under different scenarios (NASEM 2016; Weatherhead et al. 2018). Thus, the focus of Objective 2 is to address the following research questions:

- **Q6**: How are regional and global climate impacts altered through different SRM implementation scenarios and strategies under different future climate scenarios?

This, in turn, requires addressing critical gaps in existing capabilities to understand the current state of the atmosphere and climate.

In the context of near-term climate risks and SRM, priorities within these broad areas of research can be focused by identifying where rapid and high-value progress could be achieved in the following:

- Reducing uncertainty in aerosol influences on atmosphere and climate.
- Improving projections of near-term climate impacts and risk analyses with and without SRM.
- Identifying where climate-related risks and impacts are most likely to be influenced by SRM.

We have proposed a high-level framework with examples to support dialogue and further define a roadmap for modeling and analyses (Table 2) and observations (Table 3) to meet Objective 2. It includes the identification of minimum essential advances or targets for accelerating progress in Earth system models and analytics, atmosphere and climate observations, and climate research (e.g., cloud–aerosol effects, tipping events) to support requisite research activities in the defined 5-year timeframe.

**Table 2:** Proposed roadmap for Objective 2—evaluate the projected near-term impacts and risks of climate change with and without SRM (Earth system models and analyses)

R&D category	Subcategory	5-year goals*
Earth system model development	Atmospheric processes associated with SRM	• Improved representation of stratospheric chemistry and transport • Improved representation of cloud–aerosol effects in the boundary layer • Improved representation of aerosol effects on cirrus clouds
	General	• Accelerated downscaling at resolutions needed for hydrology, forestry, agriculture, health, and similar problems • Accelerated high-resolution modeling efforts • Accelerated advances in the sophistication of dynamically coupled ecosystem models • Accelerated improvements to the representation of abrupt changes and feedbacks, including permafrost, wetlands, and forest diebacks
	Technology	• Accelerated data assimilation tools and capabilities • Accelerated incorporation of machine learning in study design and analysis • Accelerated adoption of cloud computing for expanded capacity and access • Increased investment in the technology workforce
Non-model analytical tools and methods	Complex systems science	• Accelerated application of methods from other fields to climate system analysis
	Risk analysis and risk management	• Accelerated application of methods from other fields to climate risk management
	Artificial intelligence and machine learning	• Accelerated application of methods from other fields to climate processes, impacts, and risks
Studies of near-term impacts and risks with and without SRM	Global and regional climate model studies	• Corpus of model simulations of climate impacts with and without SRM for various scenarios for emissions, SRM implementation, and other climate responses configured to generate data to support analysis of impacts on hydrology, weather, terrestrial ecosystems, and ocean ecosystems with multiple global climate models (Q6) • Corpus of integrated assessment model studies of impacts with and without SRM under various scenarios to support analysis of biodiversity, human health, energy systems, infrastructure, economic productivity, and global security (Q6)
	Analog studies	• Corpus of studies of natural and anthropogenic SRM analogs (e.g., volcanic eruptions, industrial and natural emissions that influence clouds, COVID-19 lockdowns, International Maritime Organization 2020 rule for shipping and other global emissions impacting events)
	Tipping event analyses	• Methodology for analysis to identify precursor signals and potential observational metrics for major tipping points • Systematic evaluation of major near-term (e.g., 10–40 year) tipping risks (e.g., permafrost releases, forest dieback, and ice sheet collapse) • Evaluation of tipping risks most susceptible to reduction by SRM (Q6) • Suite of studies on various natural system abrupt change pathways against scenarios for SRM implementation (Q6)

^*^Specific targets will be further defined

**Table 3:** Proposed roadmap for objective 2—evaluate the near-term impacts and risks of climate change with and without SRM (observations)

R&D category	Subcategory	5-year goals*
Atmospheric observations of high relevance for aerosol-forcing effects/SRM	Stratosphere	• Baselines of key aerosol processes and populations across seasons and hemispheres and in the lower stratosphere • Sustained observational capabilities adequate to detect and monitor significant influences on solar radiation and stratospheric chemistry • Response capabilities for observations of natural system analog releases of material into the stratosphere (e.g., energetic volcanic eruptions and large wildfires (pyrocumulonimbus)) • Observations of anthropogenic analogs (e.g., aircraft and rocket plumes)
	Marine boundary layer	• Observations of background aerosol and meteorological conditions in MCB-susceptible regions (e.g., off the west coasts of the U.S ., Peru/Chile, Angola/Namibia, and Australia) • Sustained observations of marine aerosols to study anthropogenic analogs • Sustained observational capabilities adequate to detect significant influences on solar radiation
	Cirrus clouds	• Observations of background conditions in thinning-susceptible regions • Sustained observation of “clean” low-cloud condensation nuclei and high contrail-prevalent environments (e.g., Sierra Nevada and the Rockies) • Observations of anthropogenic analogs (e.g., aircraft plumes)
	Ocean surface	• Sustained aerosol, GHG, and meteorological observations at the ocean surface in targeted regions
Atmospheric composition	GHGs	• Sustained observational capabilities sufficient for flux quantification, attribution of sources, and detection of significant changes in feedbacks from natural systems
	General	• Accelerated efforts to eliminate gaps in critical observables • In situ observations to provide expanded ground-truth sources to support accelerated improvement in satellite information
Other natural systems	• Polar regions • Permafrost • Terrestrial ecosystems • Ocean ecosystems • Atlantic meridional overturning circulation • Monsoon	• Sustained observations sufficient for reducing uncertainties in major natural system feedbacks (e.g., forest dieback, permafrost, and methane clathrates) • Sustained observations sufficient for detection of key metrics identified as precursors and risk indicators for major abrupt changes (e.g., major forest collapse, ice sheet collapse, and major GHG release from permafrost)

^*^Specific targets will be further defined

### Time dependencies and critical-path activities

**Fig. 1:**
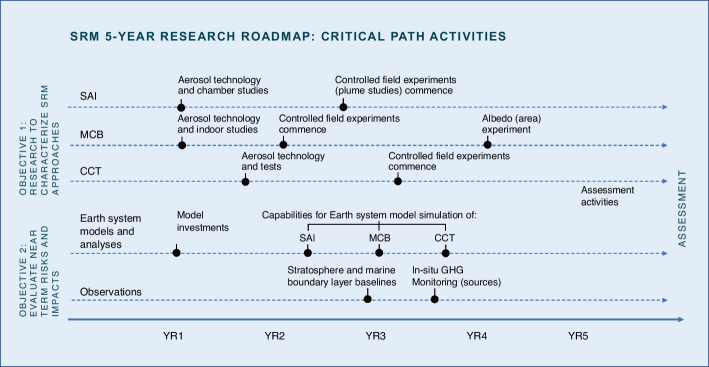
Examples of critical-path activities of an SRM 5-year roadmap by research objective

One of the most important aspects of a coordinated research effort is to deliver against the defined time horizon. A critical part of this process is identifying major time dependencies—milestones in research or capabilities development that must be reached to deliver information or capabilities required for other required research or development activities. This allows for prioritization of activities against the established timeline. We suggest several key time dependencies (Fig. 1) to deliver a 5-year assessment for near-term climate risks and SRM approaches; these are preliminary, illustrating important influences on the ability to deliver information against an explicit timeframe.

Dependencies lie in areas of activity required to produce information and/or capabilities to inform scientific assessment directly and/or as prerequisites to other required research, placing them on the “critical path” in planning. Key examples of these critical-path dependencies include, but may not be limited to, the following:

- **Controlled release experiments**: Controlled release experiments are logistically complex and more resource intensive than other research activities. For example, they require technology and take time and resources to plan, develop, and analyze. Such studies therefore present a particular challenge when trying to deliver new learning in a specified timeframe. A decision must be made early in the roadmap timeline whether an adequate assessment can be made within the period without controlled release experiments and whether required activities can commence when needed within the assessment timeline. For SRM, proposed restrictions on experiments that risk delay in their execution (ScoPEx 2021; Pidgeon et al. 2013; Gannon and Hulme 2018) should be considered in the context of the high cost of delay to timely assessment, particularly where similar studies are already undertaken for environmental protection (Voigt et al. 2021; Schumann et al. 2002; Anderson et al. 2011; Petzold et al. 2008). [see Box]
- **Earth system prediction model advancements**: Today, global Earth system prediction models do not support comprehensive representation of the atmospheric processes associated with SRM (Kravitz et al. 2020), and those treatments are rarely used for realistic simulations of the fully interactive Earth system. Until recently, except in one early instance for SAI (Tilmes et al. 2018), climate models have not included the ability to simulate controlled dispersion of aerosols as proposed for SRM. Model development and enhancement take time and are often built on advancements in modeling at higher resolutions or over smaller domains. As such, improvements in modeling capabilities need to be made in advance of when projections are needed.
- **Atmospheric baselines and monitoring capabilities**: There are substantial gaps in the characterization of the present-day composition of the atmosphere, which is needed to produce baseline simulations, improve models for research and assessment, and monitor significant changes in atmospheric composition from natural or anthropogenic sources (Cavallaro et al. 2018). Given that there is also natural variability to baseline properties, these measurements need to be conducted over multiple seasons and years for representative sampling.

**Table:** 

*A Note on Release Experiments and Proposals for Delay *
Research to assess SRM approaches requires an integrated comparison of high-resolution models and observations across a range of scales. NASEM (2021c) and others (Wood and Ackerman 2013; Dykema et al. 2014) have suggested that small-scale controlled release experiments may be important to provide critical information on processes important to SRM that are not available by other means and to test key physical processes in higher-resolution simulations, such as at the plume and (for MCB and CCT) cloud scale. They have also suggested that it is feasible to obtain this information with experiments that have negligible effects on the environment and Earth system
As proposed by SRM researchers and discussed by NASEM (2021c), small-scale experiments can be used to understand critical SRM processes that are far removed technologically or environmentally from SRM implementation and that have negligible environmental impact. These small-scale experiments would be similar in nature to release experiments that are currently undertaken for environmental research (Bulzan et al. 2010; Stokstad 2008; Pretzsch et al. 2019; Flossman et al. 2019; Tessendorf et al. 2019). In the United States and many other countries, such experiments are subject to existing regulations on physical and environmental safety. A component of any SRM research roadmap should include identifying where key uncertainties cannot be resolved through modeling and passive observations but could be addressed through small-scale release experiments. The type, mass, and scale (temporal, spatial) over which material would need to be released should be determined based on the physical science requirements of the experiment. As in other current research areas (e.g., weather modification, fuel emissions studies) the proposed release should then be assessed to assure it would have negligible impacts on climate and the environment and reviewed for compliance with existing regulations
SRM experiments at any scale have been characterized as posing non-physical (or “societal”) risks, such as a disincentive for reducing GHG emissions (sometimes referred to as a “moral hazard”). Evidence to date, however, does not support SRM research reducing incentives for GHG mitigation (Fairbrother 2016; Merk et al. 2018; Raimi et al. 2019), and, in some cases, the possibility of SRM as a component of society’s response to climate change increased support for mitigation (Merk et al. 2016; Cherry et al. 2021). Empirical research is needed to explore moral hazard and other societal dynamics associated with near-term climate risk and various responses. Similarly, evidence is needed to support assertions that delays in research associated with preferential status for inaction due to caution (sometimes referred to as “the precautionary principle”) or other non-hazard drivers of governance lead to better public welfare and environmental outcomes than the availability of more information through research

Notably, delivering against goals requires prioritizing outcomes and evaluating research plans and activities against their influence on timelines. This may require tradeoffs against consensus practices, and it may prove beneficial for smaller, more focused communities of research to move in concert in some areas.

### Development of future detailed roadmaps

From a high-level, interdisciplinary roadmap (such as that proposed here), more specific roadmaps can be developed for individual disciplines and major areas of research. These roadmaps can, in turn, support reasonably accurate cost estimates for each line of activity and inform estimates of resources required to deliver against the 5-year roadmap. They can also support the identification and creation of ongoing collaborations to deliver against each line or research, including (when warranted) multiple parallel efforts to reduce technical and execution risks and better address the magnitude of complexity (Bonvillian et al. 2019).

## Applications

Roadmaps for near-term climate and SRM research enable a variety of activities in a constructive forward path for research, cooperation, and decision-making.

### International cooperation

As climate impacts escalate, the likelihood increases that some countries or actors may attempt climate interventions, including SRM, in response to environmental and/or humanitarian threats or crises. International cooperation on research is essential to expanding and diversifying the research ecosystem, promoting equitable access to information, developing local expertise for consultation with communities, and supporting cooperative, science-based decision-making on courses of action (Biniaz and Bodansky 2020; NASEM 2021c). It is particularly critical that Global South communities are included for both adequate scientific coverage of these regions and for informed and equitable decision-making.

Multiple international assessment and scientific research coordination bodies are well-positioned to play a role in informing and/or assessing near-term climate risks and SRM. A robust but relatively narrow form of this is already underway within the Montreal Protocol, with the potential effects of SAI on the stratosphere being included in the 2022 Scientific Assessment of Ozone (The World Meteorological Organization 2022 Scientific Assessment of Ozone). A goal-oriented research roadmap would facilitate expanded participation and coordination of international and intergovernmental efforts.

### US research

The proposed approach herein was developed in the US context, where resources and technology are relatively abundant, related research is being undertaken, and a national research program in SRM has been formally recommended by a congressionally chartered scientific academy (NASEM 2021c; National Academy of Sciences n.d.; Blair 2016). A well-designed US research and assessment effort, emphasizing open science and technology access, could promote international cooperation and more effective and peaceful decision-making (Bodansky and Wanser 2021). The USA has also developed plans and/or capabilities for disaster risk management against global catastrophic threats of lower likelihood than global catastrophic climate changes (FEMA and NASA 2015, NSTC 2018, Wilcox et al. 2016).

US climate research efforts across multiple government agencies are coordinated through the U.S. Global Change Research Program (USGCRP), which has successfully delivered rigorous assessments of climate change and projected impacts on US communities and industries (Wuebbles et al. 2017; Reidmiller et al. 2018). A well-designed scientific research and assessment process managed in a similarly coordinated way might support a broad multi-agency effort executing in a focused way to deliver robust information for decision-making (NAESM 2021c). If structured around a 5-year roadmap, such as the prototype proposed here, this program could produce an effective assessment in the time-sensitive context of escalating climate threats.

## Conclusion

The latest IPCC climate assessment (IPCC 2021a) makes it clear that while GHG emission reductions are essential to avoid large amounts of future climate warming, under all scenarios considered, the Earth will still experience significant warming for at least the next few decades. This presents a high risk of escalating climate extremes and a very real risk of exceeding thresholds for environmental and societal tipping events (Drijfhout et al. 2015, Lade et al. 2020, Ritchie et al. 2021) that accelerate warming and impacts beyond humans’ capacity to mitigate them. This circumstance compels the need to simultaneously work to reduce emissions while assessing options for mitigating near-term climate risk, including SRM. Importantly, the information and capabilities available today are inadequate for these purposes (Bodansky and Biniaz 2020).

A US national research effort, such as that recommended by NAESM (2021c), could establish a model for effective governance while supporting the generation of information and development of national and international policies and monitoring capabilities for any use of SRM (Bodansky and Wanser 2021). Such a research program, built around a proposed roadmap with goals set against a defined timeframe, is essential for addressing key questions about the potential benefits and risks of SRM against the impacts of projected warming to inform decisions about climate safety. Provided that research is undertaken openly, that scientific resources are made widely available, and that scientific collaboration with experts in less developed countries is well supported, the transparency of the process and the evidence generated by research would contribute to the democratization of information, more informed and effective decision-making, and better opportunities for all people to weigh courses of action against the dire risks posed by climate change (Blicharska et al. 2017).

## Data Availability

No datasets were generated or analyzed during the current study.

## Footnotes

1 While SRM is sometimes referred to as “geoengineering,” the term may contribute to misunderstanding the purpose and nature of SRM approaches and research. Hence, consistent with NASEM (2015), SRM is used herein.
